# Effects of Fungal Probiotics on Rumen Fermentation and Microbiota in Angus Cattle

**DOI:** 10.3390/ani15182746

**Published:** 2025-09-19

**Authors:** Lijun Wang, Maolong Li, Chaoqi Liu, Xinxin Li, Ping Wang, Juan Chang, Sanjun Jin, Qingqiang Yin, Qun Zhu, Xiaowei Dang, Fushan Lu

**Affiliations:** 1 College of Animal Science and Technology, Henan Agricultural University, Zhengzhou 450046, China; wlj880626@163.com (L.W.); 15093389011@163.com (C.L.); 18790527526@163.com (X.L.); wangping850516@163.com (P.W.); changjuan2000@126.com (J.C.); 2 Henan Delin Biological Product Co., Ltd., Xinxiang 453000, China; limaolong2012@163.com (M.L.); zhuqun1991@126.com (Q.Z.); dang355@163.com (X.D.); 3 Henan PUAI Feed Co., Ltd., Zhoukou 466000, China; lfshn@126.com

**Keywords:** probiotics, Angus cattle, growth performance, rumen fermentation, rumen bacteria, rumen protozoa

## Abstract

The potential of fungal probiotics as ruminant feed additives has not been extensively studied. This study aimed to evaluate the effect of *A. oryzae* and *T. longibrachiatum* supplementation on Angus cattle during the early stages of fattening. In this study, 80 Angus cattle aged approximately 9~10 months (40 males and 40 females), with an average initial body weight (BW) of 276.46 ± 27.92 kg, were randomly assigned to four groups. Each group included 4 replicates (2 replicates of males and 2 replicates of females). Each replicate contained 5 male or 5 female Angus cattle. Cattle in the control group received a total mixed ration (TMR) without additives, while those in test groups 1, 2, and 3 received a TMR supplemented with complex probiotics (CPs) at 1.0, 2.0, and 3.0 g·kg^−1^ of feed dry matter (DM), respectively. The adaptation and experimental periods were 7 and 60 days, respectively. Compared with those in the control group, the apparent digestibility of ether extract, calcium, and acid detergent fiber (ADF) was higher in test group 3. Test group 2 exhibited increased apparent digestibility of hemicellulose. Meanwhile, test group 3 exhibited increased levels of acetate, propionate, butyrate, and total volatile fatty acids and decreased pH. CPs increased the abundance of the *SR1* phylum, *Solibacillus*, *Lysinibacillus*, and *Planococcaceae_Bacillus* and decreased the proportions of *Lactococcus*, *Ruminococcus*, and *Ophryoscolex*. *Solibacillus* was associated with the apparent digestibility of CP, Ca, and ADF. *Planococcaceae_Bacillus* was associated with increased apparent digestibility of CP, ADF, and hemicellulose. This suggests that CPs improve crude protein and cellulose digestion by increasing the proportions of *Solibacillus* and *Planococcaceae_Bacillus*. Therefore, the optimal CP dietary supplementation dose for Angus cattle was 3 g·kg^−1^ of DM.

## Simple Summary

The fattening period, a critical stage in beef cattle farming, is characterized by an increase in growth and body weight. Probiotics enhance rumen fermentation, improve nutrient absorption, and increase daily weight gain in ruminants. However, the potential beneficial effects of specific fungal probiotics in ruminants, especially when used in combination, have not been examined. *Aspergillus oryzae* and *Trichoderma longibrachiatum* exert beneficial effects on rumen function and fiber digestion. However, the effects of the combination of *A. oryzae* and *T. longibrachiatum* as a feed additive have not been studied. This study demonstrated that complex probiotic supplementation significantly improved the apparent digestibility of key nutrients (such as crude protein, fats, and fibers). Furthermore, it improved rumen fermentation by augmenting the production of volatile fatty acids and decreasing pH levels. Simultaneously, it positively altered the rumen bacterial community by promoting bacteria linked to enhanced digestion. These findings suggest that fungal probiotics can effectively support rumen health and digestion during the fattening period.

## 1. Introduction

Angus cattle undergo transitions in physiological status, dietary structure, and growth requirements during the initial fattening phase. Feed intake gradually increases, while appetite is dependent on dietary changes and digestive disturbances during the fattening phase [1]. Thus, intestinal health must be regulated to ensure feed intake stability. Supplementation with compound probiotics (CPs) is reported to support a smooth transition through this critical period by modulating gut microbiota, enhancing digestion, and strengthening immunity [2,3]. This approach lays the groundwork for subsequent efficient weight gain and meat quality improvement in later fattening stages.

Probiotics are live microorganisms that provide health advantages to the host by settling in the intestinal tract and adjusting the balance of gut microbiota [4]. The following properties of probiotics have piqued the interest of the scientific community: (1) enhancing animal growth performance; (2) inhibiting pathogenic microorganism colonization; (3) maintaining digestive tract microflora homeostasis [5,6,7]. Commercially available probiotic preparations comprise bacterial, fungal, and yeast strains [8]. Historical applications in animal husbandry have focused on bacterial and yeast probiotics. Feed supplementation of ruminants with fungal probiotics has not been explored.

Fungal probiotics are one of the emerging areas [9]. Previous studies reported that *Aspergillus oryzae* is a prevalent probiotic that is extensively used as a feed additive in ruminant farming [10,11]. *A. oryzae* improves the breakdown of feed dry matter, alleviates inflammation, and increases the energy contribution of total volatile fatty acids (TVFAs) in the rumen [12]. Additionally, *A. oryzae* produces amylases and proteases during the different phases of growth [13]. *T. longibrachiatum* can produce exogenous fibrolytic enzymes to accelerate fiber digestion by enhancing the attachment of ruminal microorganisms [14]. Cellulase secreted by *T. longibrachiatum* can decompose cellulose into small-molecule carbohydrates, which are available for *A. oryzae* utilization. Subsequently, the protease produced by *A. oryzae* can break down proteins to generate nitrogen sources, such as amino acids, which may be absorbed by *T. longibrachiatum*. Thus, these two fungi exhibit a symbiotic relationship [15]. Enzymes can be used to stimulate ruminal microbes to generate increased amounts of microbial biomass, which will provide polysaccharide enzymes to digest feed. One study demonstrated that feed enzymes in dairy cows increased rumen digestion and the flow of microbial protein from the rumen [16].

Previous studies have investigated the functions of *A. oryzae* and *T. longibrachiatum*. However, limited studies have examined the effects of the combination of *A. oryzae* and *T. longibrachiatum*. This study aimed to explore the impact of combined compound probiotics on growth performance, apparent nutrient digestibility, and microbial composition in the rumen of Angus cattle.

## 2. Materials and Methods

### 2.1. Microbial Feed Additive Preparation

*A. oryzae* (CGMCC5817), which was extracted from a cow’s rumen and identified using 26S rDNA, was obtained from the China General Microbiological Culture Collection Center (CGMCC). *T. longibrachiatum* (CGMCC 3.1029) was also obtained from CGMCC. The probiotic powder was prepared by incubating them following the established protocols [17]. According to earlier findings from our lab using response surface regression design in vitro. CPs were prepared by mixing *A. oryzae* and *T. longibrachiatum* in the ratio 50:50 each with 1.5 × 10^9^ colony-forming units (CFU)/g of probiotic powder.

### 2.2. Experimental Design and Animal Management

All animal experiments were approved by the Animal Care and Use Committee of Henan Agricultural University (Approval number: HENAU-2021-025). The experiments were conducted at the Qixian Animal Husbandry Station of Henan Agricultural University, located in Kaifeng, Henan, China.

This study selected 80 Angus cattle aged 9~10 months (40 males and 40 females) with an average initial body weight (BW) of 276.46 ± 27.92 kg, which were randomly assigned to four groups. Each group included 4 replicates (2 replicates of males and 2 replicates of females). Each replicate contained 5 male or 5 female Angus cattle. To ensure controlled conditions, male and female Angus cattle within each replicate were housed separately in individual pens. The experimental diet for different groups was as follows: control group: total mixed ration (TMR); test group 1: TMR + CPs (1.0 g·kg^−1^); test group 2: TMR + CPs (2.0 g·kg^−1^); test group 3: TMR + CPs (3.0 g·kg^−1^). During the study period, Angus cattle were fed twice daily at 8:00 AM and 4:00 PM. The amounts of feed offered to each pen and leftover feed were daily recorded. All animals had access to clean, fresh water ad libitum. The study included a 7 days adaptation period and a 60 days experimental period. The diets were formulated to meet or exceed NRC (2000) requirements for growing and finishing Angus cattle. Table 1 shows the ingredients and calculated nutrient composition of the diets.

### 2.3. Growth Performance

The BW of Angus cattle was measured before the morning feeding at the beginning and at the end of the trial to determine average daily gain (ADG). During the study period, the weight of the feed provided and that of the leftover feed for each replicate were documented daily to calculate the average daily feed intake (ADFI). The ADG, ADFI, and feed conversion ratio (FCR) were calculated as follows:

ADG = (finish weight − start weight)/number of experimental days

ADFI = (provided feed amount − residual feed amount)/number of Angus cattle

FCR = average daily feed intake/average daily gain

### 2.4. Sample Collection

Approximately 100 mL of rumen fluid was aseptically collected from one Angus cattle per replicate (each selected based on similar live weight) using a gastric tube before morning feeding. The samples were immediately stored at −20 °C for subsequent analyses of pH, volatile fatty acids (VFAs), and ammonia-N (NH3-N) content. The rumen fluids (approximately 3 mL) were transferred to a sterile tube, snap-frozen in liquid nitrogen, and stored in a −80 °C freezer for rumen microbiota analysis.

### 2.5. Apparent Nutrient Digestibility Determination

The feces were sampled daily at 08:00 AM and 5:00 PM. From Day 57 to Day 59 of the experiment, the total fecal samples were collected from four Angus cattle in each group, weighed, mixed thoroughly, and divided into two portions. One portion was stored, while the other portion was treated with 10% sulfuric acid solution (20 mL acid per 100 g feces) and stored at −20 °C for later analysis. Some fecal samples were dried at 65 °C for 24 h and mashed to determine apparent nutrient digestibility. The levels of dry matter (DM), crude protein (CP), ether extract (EE), Ca, P, neutral detergent fiber (NDF), acid detergent fiber (ADF), cellulose, and hemicellulose in feces and diets were determined following the previously reported protocols [18,19]. Acid-insoluble ash (AIA) was determined following the methods of Van Keulen and Young (1977) [20]. Apparent digestibility of nutrients was calculated as follows:

Apparent digestibility (%) = 100 − (diet AIA%/fecal AIA %) × (fecal nutrient%/diet nutrient%) × 100

### 2.6. Rumen Fermentation Parameters

The ruminal VFAs, such as acetate, propionate, butyrate, and isobutyrate, were assessed using an ion chromatograph (ICS150, Sykam Corporation, Munich, Germany). Meanwhile, the rumen fluid level of NH_3_-N was assessed using the phenol–hypochlorite colorimetric method, following the protocols of Broderick and Kang (1980) [21]. The rumen pH was assessed with a pH meter (PHB-4; Shanghai Leici Co., Ltd., Shanghai, China).

### 2.7. Microbiota Analysis in Rumen Fluid

The rumen fluid samples were retrieved from the −80 °C freezer for microbial DNA sequencing, which was performed by Shanghai Paisennuo Biotechnology Co., Ltd., Shanghai, China. The V3–V4 regions of the bacterial 16S rRNA or the V4–V5 regions of ciliate 18S rRNA were sequenced using the Illumina MiSeq platform. Primer removal, mass filtering, denoising, stitching, and de-embedding were performed using the DADA2 method in the QIIME2 platform. The sequences obtained were termed application sequence variants (ASVs) [22]. ASVs were classified using the Naive Bayes consensus taxonomy classifier and aligned to databases with the classify-sklearn plugin (https://github.com/qiime2/q2-feature-classifier, accessed on 22 March 2021) and the Greengenes version 13.8 database (http://greengenes.lbl.gov, accessed on 22 March 2021) [23,24].

### 2.8. Statistical Analysis

Growth performance, apparent nutrient digestibility, fermentation parameters, and enzyme activity of the rumen were analyzed using one-way analysis of variance IBM SPSS Statistics Version 26 (IBM SPSS Statistics, IBM Corporation, Armonk, NY, USA). The statistical model employed was the following:

Yij = μ + Ti + εij,

where Yij, represents the j-th observation in the i-th group, μ is the overall mean, Ti is the treatment effect for the i-th group, and εij is the error term. If the ANOVA results indicated significant differences between the treatment groups, Duncan’s Multiple Range Test was used for multiple comparisons to determine which specific groups showed significant differences. *p* < 0.05 is considered significant, 0.05 ≤ *p* ≤ 0.10 is considered a trend. The results are expressed as mean ± standard deviation.

The correlation between environmental factors and rumen bacteria or ciliates (with relative abundance > 0.5) was analyzed using the Spearman correlation analysis (Spearman coefficient). All correlation heat maps and hierarchical clusters were prepared with the CRAN “pheatmap” package of R Studio (v1.0.13).

## 3. Results

### 3.1. Effect of CPs on the Growth Performance and Feed Efficiency of Angus Cattle

The growth performance data of Angus cattle are shown in Table 2. The difference in growth performance between the control and test groups was not significant (*p* > 0.05).

### 3.2. Effects of CPs on Apparent Nutrient Digestibility

As shown in Table 3, the apparent digestibility of EE and ADF in test group 3 was significantly higher than that in the control group (*p* < 0.05). Meanwhile, the apparent digestibility of Ca in test group 3 was significantly higher than that in test groups 1 and 2 (*p* < 0.05) but was not significantly different from that in the control group (*p* > 0.05). Additionally, the apparent digestibility of NDF in test group 1 and that of hemicellulose in test group 2 were significantly higher than those in the control group (*p* < 0.05).

### 3.3. Effects of CPs on Ruminal Fermentation Parameters

Table 4 shows the rumen fermentation parameters. The concentrations of propionate in test group 3 were significantly higher than those in test groups 1 and 2 (*p* < 0.05) but were not significantly different from those in the control group (*p* > 0.05). Butyric and total VFAs in test group 3 tended to be higher than those in test groups 1 and 2 (0.05 ≤ *p* ≤ 0.10).

### 3.4. Effects of CPs on Bacterial Abundances at the Phylum Level

Cattle in test group 3 exhibited enhanced growth performance, apparent nutrient digestibility, and ruminal fermentation parameters. Thus, further experiments were performed with the control group and test group 3.

**Figure 1:**
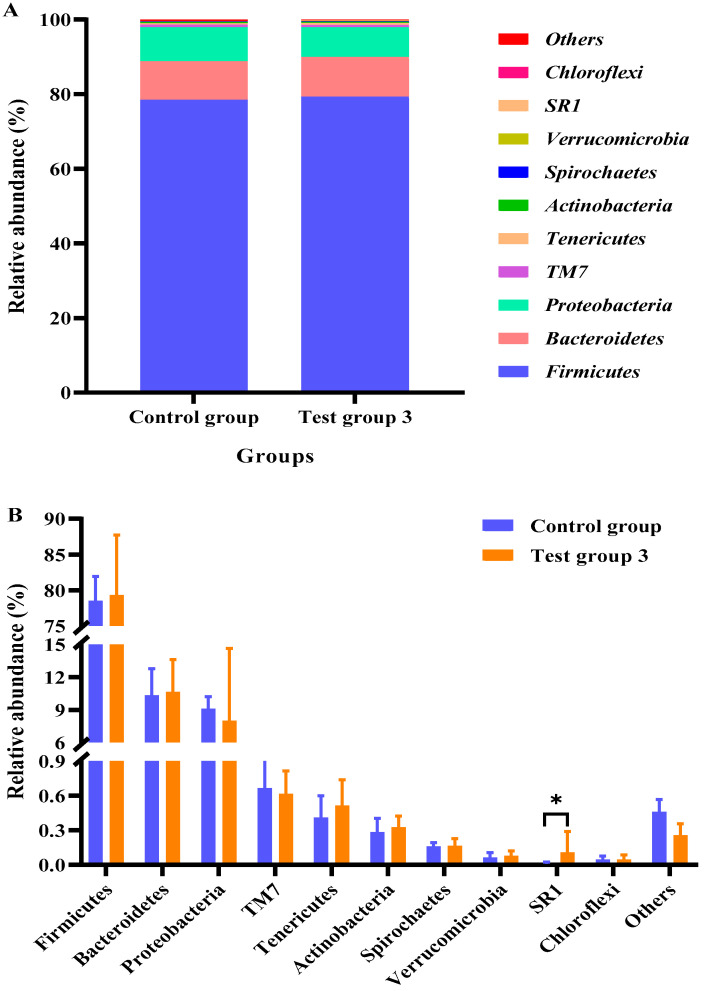
Effects of compound probiotics (CPs) on bacterial abundances at the phylum level in the rumen fluid of Angus cattle (**A**); Statistical analysis of the top ten phyla (**B**). * *p* < 0.05.

Figure 1 shows the ten most abundant microbial phyla. At the phylum level, the rumen bacterial community comprised *Firmicutes*, *Bacteroidota*, and *Proteobacteria*, which accounted for approximately 95% of the top 10 phyla. The abundances of these bacterial phyla were similar between the control group and test group 3 (*p* > 0.05). Compared with those in the control group, the relative abundances of *Firmicutes*, *Bacteroidetes*, *Tenericutes, Actinobacteria*, *Spirochaetes*, and *Verrucomicrobia* were higher, and the relative abundances of *Proteobacteria*, *TM7*, and *Chloroflexi* were lower in test group 3 (*p* < 0.05). The relative abundance of *SR1* in test group 3 was higher than that in the control group (*p* < 0.05).

### 3.5. Effects of CPs on Bacterial Abundances at the Genus Level

**Figure 2:**
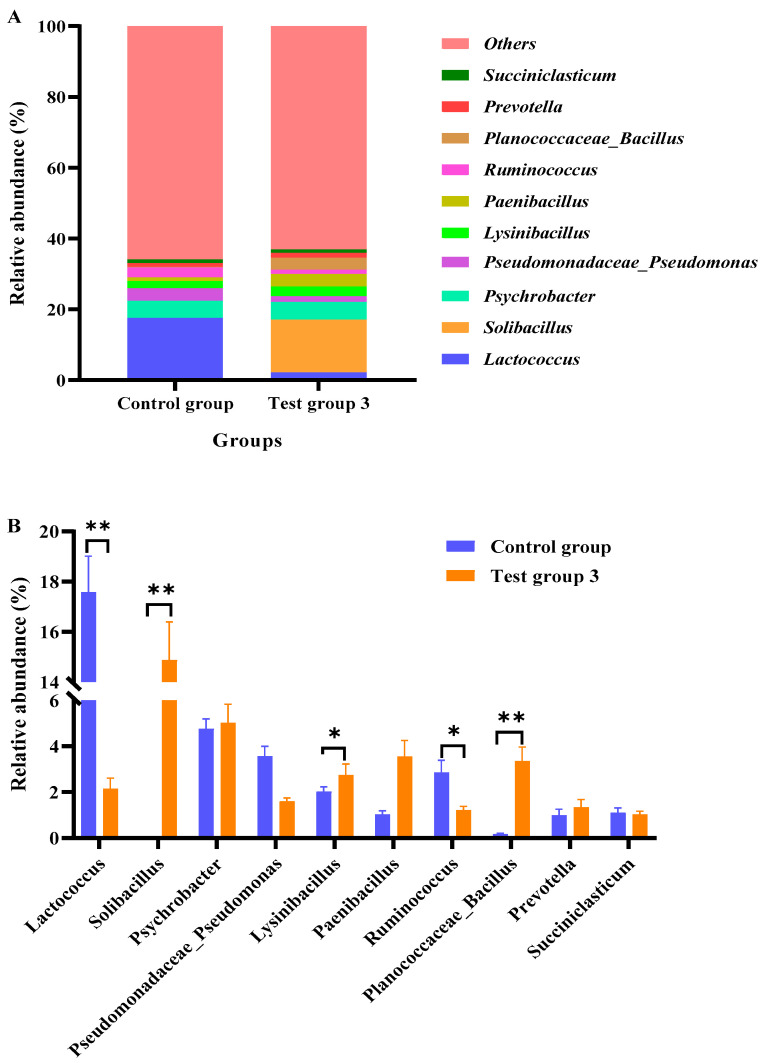
Effects of compound probiotics (CPs) on bacterial abundances at the genus level in the rumen fluid of Angus cattle (**A**); Statistical analysis of the top 10 genera (**B**). * *p* < 0.05; ** *p* < 0.01.

Figure 2 shows the top ten genera in the rumen fluid of Angus cattle. The rumen fluid abundances of *Solibacillus*, *Lysinibacillus*, and *Planococcaceae_Bacillus* in test group 3 were higher than those in the control group (*p* < 0.05). In contrast, the relative abundances of *Lactococcus* and *Ruminococcus* in test group 3 were lower than those in the control group (*p* < 0.05). Compared with those in the control group, the relative abundances of *Paenibacillus* and *Prevotella* were non-significantly higher, and the relative abundances of *Pseudomonadaceae_Pseudomonas* were non-significantly lower in test group 3 (*p* > 0.05).

### 3.6. Correlation of Bacterial Communities with Growth Performance, Apparent Nutrient Digestibility, and Fermentation Parameters

**Figure 3:**
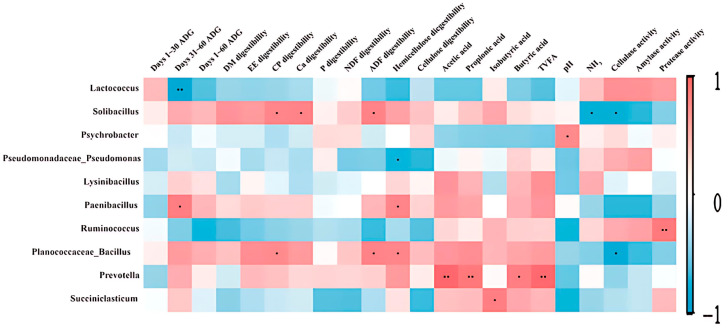
Correlation between rumen bacterial abundance and the growth, nutrient digestion, and fermentation characteristics of Angus cattle. Data of only significant correlations (r > 0.60 or r < −0.60 and *p*-value < 0.05) and bacteria with abundances > 0.5% are presented. Positive (coefficient close to 1) and negative (coefficient close to −1) correlations between the examined traits and bacterial abundances are indicated in red and blue colors, respectively. ^•^ *p* < 0.05; ^••^ *p* < 0.01.

Pearson’s correlation coefficient analysis revealed that the abundances of the top 10 genera were closely associated with growth performance, apparent nutrient digestibility, and fermentation parameters (Figure 3). The abundance of *Solibacillus* was significantly and positively correlated with the apparent digestibility of CP, Ca, and ADF and negatively correlated with NH_3_-N and cellulase activity (*p* < 0.05). Meanwhile, the abundance of *Paenibacillus* was significantly and positively correlated with ADG on days 31–60 and apparent digestibility of hemicellulose (*p* < 0.05). Additionally, the abundance of *Planococcaceae_Bacillus* was significantly and positively correlated with the apparent digestibility of CP, ADF, and hemicellulose (*p* < 0.05) and negatively correlated with cellulase activity (*p* < 0.05). *Prevotella* abundance was significantly and positively correlated with acetate, propionate, butyrate, and TVFA levels (*p* < 0.05). *Succiniclasticum* abundance was significantly and positively correlated with isobutyric acid levels (*p* < 0.05).

### 3.7. Effects of CPs on the Abundances of Ciliates

**Figure 4:**
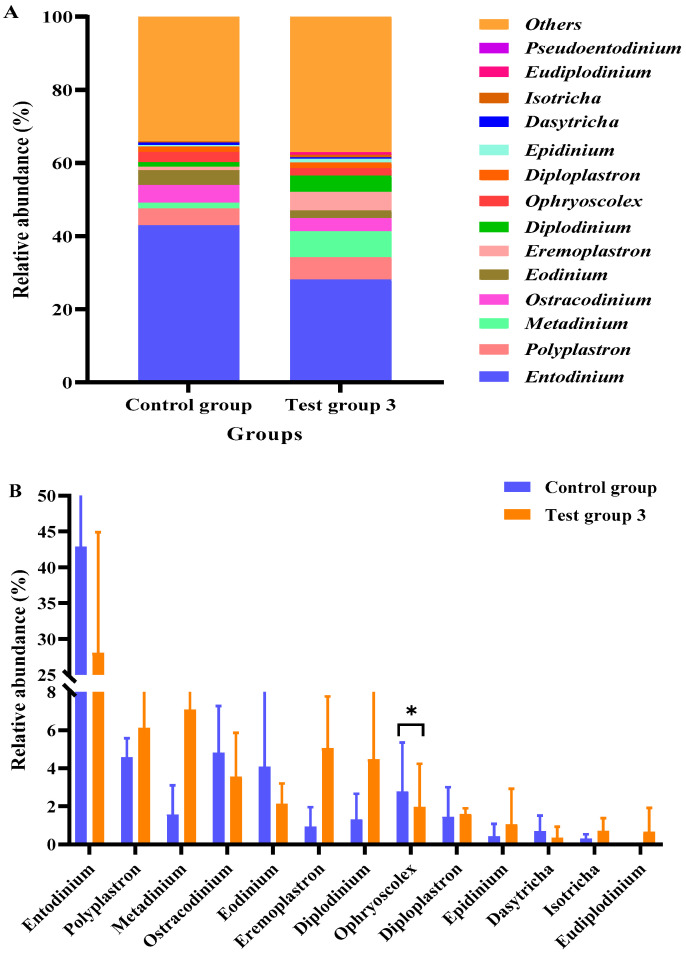
Effects of compound probiotics (CPs) on the abundances of ciliate genera in the rumen fluid of Angus cattle (**A**); statistical analysis of the relative abundance of ciliate genera (**B**). * *p* < 0.05.

As shown in Figure 4, the relative abundance of *Ophryoscolex* in test group 3 was significantly lower than that in the control group (*p* < 0.05). Compared with those in the control group, the relative abundances of *Polyplastron*, *Metadinium*, *Eremoplastron*, *Diplodinium*, *Epidinium*, *Isotricha*, and *Eudiplodinium* were non-significantly higher, and the relative abundances of *Entodinium*, *Ostracodinium*, *Eodinium*, *Diploplastron*, and *Dasytricha* were non-significantly lower in test group 3 (*p* > 0.05).

### 3.8. Correlation of Ciliate Communities with Growth Performance, Apparent Nutrient Digestibility, and Fermentation Parameters

**Figure 5:**
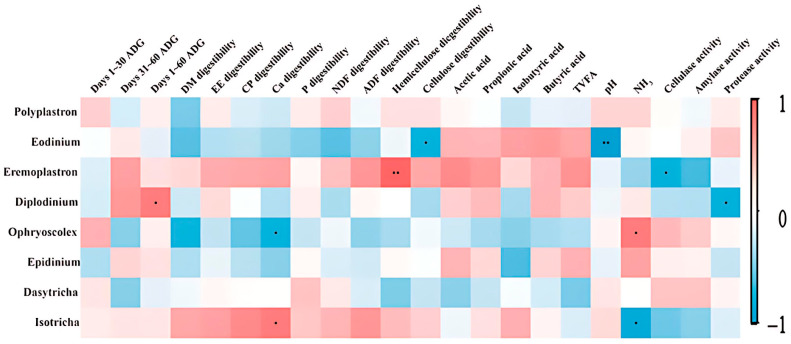
Correlations of rumen bacteria with the growth, apparent nutrient digestibility, and fermentation characteristics of Angus cattle. Data of only significant correlations (r > 0.60 or r < −0.60 and *p* < 0.05) and bacteria with abundances > 0.5% are presented. Positive (coefficient close to 1) and negative (coefficient close to −1) correlations between the examined traits and bacterial abundances are indicated in red and blue colors, respectively. ^•^ *p* < 0.05; ^••^ *p* < 0.01.

Figure 5 shows a correlation heat map illustrating the relationship of rumen ciliates at the genus level with the growth performance, apparent nutrient digestibility, and fermentation parameters. The abundances of *Eremoplastron*, *Diplodinium*, *Ophryoscolex*, and *Isotricha* were significantly correlated with hemicellulose digestibility, ADG on days 1–60, NH_3_-N concentration, and Ca digestibility (*p* < 0.05), respectively. *Eodinium* abundance was significantly and negatively correlated with cellulose digestibility and rumen pH (*p* < 0.05).

## 4. Discussion

In this study, the ADFI, ADG, and FCR were not significantly different between the groups. Previous studies have reported that the dietary supplementation of CPs did not significantly affect the growth performance of Holstein calves [25,26], consistent with our study. However, Podversich et al. (2023) [27] revealed that *A. oryzae* supplementation significantly improved the feed efficiency of Angus cattle. Additionally, Tricarico et al. (2007) [28] reported that the dietary supplementation of *A. oryzae* increased ADG. These differential results can be attributed to various factors, including the health status, the level of stress in cattle, and the exposure to intestinal pathogens during rearing.

The rumen comprises a diverse and intricate microbial ecosystem. The microbial community can promote the breakdown of substances that are difficult to digest (such as cellulose) by ruminants through fermentation. Additionally, these microbes synthesize proteins to provide nutrition for the host. In the current study, the apparent digestibility of NDF surpassed 80% following the administration of a composite probiotic (*A. oryzae* and *T. longibrachiatum*). Conversely, prior in vitro fermentation studies have indicated that supplementation with *A. oryzae* alone achieved an NDF digestibility marginally exceeding 40% [10]. Consequently, the utilization of a composite probiotic incorporating both *A. oryzae* and *T. longibrachiatum* markedly improved the apparent digestibility of NDF in ruminants, in comparison to the use of Aspergillus oryzae in isolation. In the present study, CPs enhanced the apparent digestibility of calcium. Probiotics alter the gut microbiota structure and gene activity to promote calcium absorption [29]. The gut microbiome enhances calcium absorption, retaining calcium and improving bone health [30]. In particular, probiotics markedly enhance the serum calcium levels [31]. Probiotics can alter the microbial ecosystem in the rumen to boost apparent nutrient digestibility and feed efficiency. These findings indicate that CPs can improve the apparent nutrient digestibility of cattle.

Rumen fluid pH is an indicator of the internal environment, health, and fermentation status of the rumen. In the present study, CPs did not affect the pH of ruminal fluid. Consistently, two previous studies have reported that the dietary supplementation of *A. oryzae* [32] or *Trichoderma reesei* and *A. oryzae* did not alter the rumen fluid pH [33]. The rumen fluid pH reported in this study was higher than that reported in the two previous studies. This may be because the Angus cattle in this study were fed on a diet with increased roughage content. One study reported that yeast supplementation increased the rumen pH and decreased the levels of ammonia and lactate, and enhanced the levels of VFAs, especially acetate and propionate. The concentrations of VFAs in this study are similar to those reported by Várhidi et al. (2022) [34]. The generation of VFAs and lactic acid in the rumen can decrease the pH [35]. In this study, CPs increased the pH and VFAs in the rumen, which may be due to the CP-induced downregulation of lactic acid [36]. However, further studies are needed to confirm this observation. CPs upregulated the levels of acetate and propionate. This is because *A. oryzae* and *T. longibrachiatum* promote the decomposition of cellulose, establishing conducive environments for the fermentative production of acetic acid and propionic acid [37,38]. In this study, dietary CP supplementation promoted the synthesis of acetate and propionate, which are the main energy sources for the rumen wall. These findings suggest that CPs improved rumen fermentation.

In this study, the microbial community of Angus cattle predominantly comprised *Firmicutes*, *Bacteroidetes*, and *Proteobacteria* at the phylum level in all groups. These findings are consistent with those of previous studies [39,40]. However, the abundance of the phylum *SR1* in test group 3 was higher than that in the control group. Recent studies have reported that the relative abundance of *SR1* in Angus cattle is significantly increased under free-range grass-fed conditions [41]. This suggests that *SR1* may promote fiber digestion. Further studies are needed to understand the roles of *SR1* and its association with apparent digestibility in Angus cattle. The predominant genera in Angus cattle were *Lactococcus*, *Solibacillus*, *Psychrobacter*, *Pseudomonadaceae_Pseudomonas*, and *Lysinibacillus*. CPs significantly decreased the abundance of *Lactococcus*. *Lactococcus* is reported to be negatively correlated with acetate, propionate, valerate, and TVFA [42]. This can explain the upregulation of acetate, propionate, and TVFA. CPs also increased the abundance of *Solibacillus*, which is reported to produce casease. During restricted resource periods, the relative abundance of *Solibacillus* increases in buffalo rumen [43]. *Solibacillus* in goats and cattle has been associated with decreased forage intake, potentially as a response to varied diets [44,45]. Consistently, *Solibacillus* was significantly and positively correlated with crude protein and ADF digestibility in this study. CPs supplementation increased the relative abundance of *Lysinibacillus*, which exhibits potent enzymatic capabilities [46]. Thus, CPs supplementation may enhance the enzymatic degradation of nutrients by increasing *Lysinibacillus* abundance. *Ruminococcus* is a primary cellulolytic bacterium in the rumen [47]. However, *Ruminococcus gnavus* produces and releases glucorhamnan, a complex carbohydrate composed of a rhamnose backbone with glucose branches, which can effectively stimulate dendritic cells to release inflammatory cytokines (TNF-α) [48]. In this study, CPs supplementation decreased the relative abundance of *Ruminococcus*, which alleviates inflammation. The relative abundance of *Planococcaceae* increases at the peak of cellulose degradation [49]. Therefore, we speculate that *Planococcaceae_Bacillus* can degrade fiber. This is consistent with the significant positive correlation between *Planococcaceae_Bacillus* and the degradation rates of ADF and hemicellulose. *Prevotella*, a resident bacterium in the rumen, can degrade and utilize starch to produce VFAs. Thus, CPs can increase carbohydrate digestibility by modulating *Prevotella* abundance in the rumen [50]. Consistently, *Prevotella* abundance was positively correlated with the contents of acetate, propionate, and TVFAs.

Ciliates are common protozoans in ruminants. The abundance of ciliates is lower than that of bacteria. However, the volume occupied by ciliates is similar to that occupied by bacteria owing to their large size [51]. Ciliates are involved in rumen fermentation, feed digestion, and generation of secondary fermentation products [52]. In addition to engulfing and utilizing starch granules and insoluble protein, ciliates can phagocytose microbial cells. In this study, the major ciliates identified in the rumen were *Entodinium*, *Polyplastron*, *Metadinium*, and *Ostracodinium*, which is consistent with the findings of Abrar et al. (2016) [53]. *Ophryoscolex*, a rumen ciliate, ferments starch, resulting in the formation of acetate, butyrate, and lactate along with CO_2_ and H_2_ [54]. In the presence of the same amounts of starch and casein as substrates, *Ophryoscolex* that utilizes casein produced increased levels of NH_3_ [54]. This explains the positive correlation between *Ophryoscolex* and NH_3_. Liu et al. (2023) [55] demonstrated that *Ophryoscolex* abundance was positively correlated with methane emission. Thus, CPs used in this study can potentially reduce methane emissions. *Eremoplastron* abundance is reported to be associated with hemicellulose digestibility [56]. CPs supplementation increased *Eremoplastron* abundance and consequently enhanced nutrient digestibility. Williams et al. (1991) [56] revealed a negative correlation between *Eremoplastron* abundance in the rumen fluid of Angus cattle and cellulase activity.

## 5. Conclusions

This study demonstrated that the optimal supplementation dose of CPs for Angus cattle fed on TMR diets was 3 g·kg^−1^. At this dose, CPs increased daily weight gain, enhanced apparent nutrient digestibility, and modified ruminal fermentation parameters. Additionally, CPs increased the relative abundances of *Solibacillus, Lysinibacillus, Planococcaceae_Bacillus* and decreased the abundance of *Ophryoscolex* at the genus level. The findings of this study suggest the potential benefits of CPs in ruminant farming. However, the underlying mechanisms by which CPs modulate the rumen microbiota and metabolism in Angus cattle require further investigation through subsequent experiments.

## Figures and Tables

**Table 1:** Composition and nutrient levels of TMR (%, dry matter basis, DM).

TMR Composition	Content (%)	Nutrient Levels	Content (%)
Corn meal	15.00	Dry matter	89.12
Wheat bran	3.00	Crude protein	9.90
DDGS ^1^	2.00	Ether extract	2.05
Soybean meal	2.00	Neutral detergent fiber	55.56
Cottonseed meal	2.00	Acid detergent fiber	22.74
Rice straw	18.50	Calcium	0.55
Alfalfa	18.50	Total phosphorus	0.26
Corn silage	37.00	Net energy/(MJ/kg)	9.56
Limestone	0.30		
CaHPO_4_	0.20		
NaCl	0.25		
NaHCO_3_	0.25		
Premix ^2^	1.00		
Total	100.00		

Notes: ^1^ DDGS: Distillers Dried Grains with Solubles. ^2^ The premix comprised the following components (per kg of TMR): vitamin A: 3000 IU; vitamin D: 3800 IU; vitamin E: 8 IU; vitamin K: 32 mg; Cu (as copper sulfate): 7 mg; Fe (as ferrous sulfate): 130 mg; Mn (as manganese sulfate): 30 mg; Zn (as zinc sulfate): 30 mg; Se (as sodium selenite): 0.077 mg; Co (as cobalt chloride): 0.31 mg; I (as potassium iodide): 0.256 mg. Net energy values were calculated from feed samples using the NRC (2000) equations, Net energy = Net Energy for Maintenance (NEm) + Net Energy for Gain (NEg) = ∑(Proportion of each feed ingredient × NEm of the respective feed ingredient) + ∑(Proportion of each feed ingredient × NEg of the respective feed ingredient). While the other nutrient levels were measured.

**Table 2:** Effect of compound probiotics (CPs) on the growth performance and feed efficiency of Angus cattle.

Items	Control Group	Test Group 1	Test Group 2	Test Group 3	*p*-Value
ADFI (kg/d)	8.56 ± 0.28	8.60 ± 0.07	8.57 ± 0.33	8.85 ± 0.21	0.267
ADG (kg/d)	0.94 ± 0.07	0.97 ± 0.12	0.93 ± 0.13	1.03 ± 0.02	0.469
FCR	9.19 ± 0.92	8.96 ± 1.14	9.35 ± 1.62	8.61 ± 0.10	0.698

Notes: Values in the same row without superscript letters indicate non-significant differences (*p* > 0.05). ADG: average daily gain; ADFI: average daily feed intake; FCR: feed conversion ratio. Control group: total mixed ration (TMR); Test group 1: TMR + CPs (1.0 g·kg^−1^); Test group 2: TMR + CPs (2.0 g·kg^−1^); Test group 3: TMR + CPs (3.0 g·kg^−1^).

**Table 3:** Effects of compound probiotics (CPs) on apparent nutrient digestibility in Angus cattle (%).

Items	Control Group	Test Group 1	Test Group 2	Test Group 3	*p*-Value
DM (%)	77.22 ± 1.15	76.25 ± 1.30	76.27 ± 2.15	77.87 ± 1.38	0.40
EE (%)	74.92 ± 1.18 ^b^	66.36 ± 3.17 ^c^	73.09 ± 2.08 ^b^	81.12 ± 1.57 ^a^	<0.01
CP (%)	76.66 ± 1.07	77.06 ± 1.02	76.09 ± 2.20	78.34 ± 0.93	0.19
Ca (%)	51.16 ± 3.43 ^ab^	50.16 ± 1.78 ^b^	47.46 ± 5.47 ^b^	55.90 ± 2.01 ^a^	0.03
P (%)	68.48 ± 2.52	68.10 ± 2.19	67.51 ± 2.36	69.95 ± 1.20	0.44
NDF (%)	86.02 ± 1.30 ^b^	87.82 ± 0.86 ^a^	85.79 ± 0.76 ^b^	87.39 ± 1.31 ^ab^	0.05
ADF (%)	73.06 ± 3.57 ^b^	76.98 ± 0.80 ^ab^	74.07 ± 2.99 ^b^	79.86 ± 1.78 ^a^	0.01
Hemicellulose (%)	95.18 ± 0.36 ^b^	95.46 ± 0.90 ^ab^	96.48 ± 0.99 ^a^	95.88 ± 0.12 ^ab^	0.05
Cellulose (%)	80.99 ± 2.46	83.26 ± 0.43	82.00 ± 1.53	84.16 ± 2.68	0.17

Notes: Values in the same row with the same lowercase letter superscripts or without letters indicate non-significant differences (*p* > 0.05). Values with different lowercase superscript letters indicate significant differences (*p* < 0.05). DM: Dry matter; EE: ether extract; CP: Crude protein; NDF: neutral detergent fiber; ADF: acid detergent fiber. Control group: total mixed ration (TMR); Test group 1: TMR + CPs (1.0 g·kg^−1^); Test group 2: TMR + CPs (2.0 g·kg^−1^); Test group 3: TMR + CPs (3.0 g·kg^−1^).

**Table 4:** Effects of compound probiotics (CPs) on rumen fermentation parameters of Angus cattle.

Items	Control Group	Test Group 1	Test Group 2	Test Group 3	*p*-Value
pH	8.19 ± 0.32	7.91 ± 0.13	8.24 ± 0.09	8.30 ± 0.29	0.13
NH_3_-N (mg/dL)	7.60 ± 1.60	5.85 ± 1.62	5.24 ± 1.81	6.10 ± 2.19	0.34
Acetic (mg/mL)	2.01 ± 0.65	1.80 ± 0.40	1.92 ± 0.28	2.56 ± 0.30	0.12
Propionic (mg/mL)	0.18 ± 0.14 ^ab^	0.13 ± 0.07 ^b^	0.15 ± 0.05 ^b^	0.34 ± 0.14 ^a^	0.04
Isobutyric (mg/mL)	0.08 ± 0.05	0.08 ± 0.04	0.04 ± 0.01	0.07 ± 0.03	0.25
Butyric (mg/mL)	0.12 ± 0.12 ^ab^	0.06 ± 0.04 ^b^	0.06 ± 0.01 ^b^	0.20 ± 0.08 ^a^	0.05
Total VFA/(mg/mL)	2.40 ± 0.92 ^ab^	2.07 ± 0.53 ^b^	2.16 ± 0.33 ^b^	3.17 ± 0.49 ^a^	0.06
Cellulase activity (U/mL)	1.77 ± 0.86	2.34 ± 1.77	2.34 ± 2.17	3.65 ± 0.21	0.46
Amylase activity (U/mL)	0.11 ± 0.03	0.13 ± 0.09	0.12 ± 0.07	0.06 ± 0.02	0.42
Protease activity (U/mL)	3.24 ± 1.92	4.36 ± 0.28	3.80 ± 1.07	5.21 ± 1.25	0.21

Note: Values in the same row with the same lowercase superscript letters or without letters indicate non-significant differences (*p* > 0.05). Values with different lowercase superscript letters indicate significant differences (*p* < 0.05). Control group: total mixed ration (TMR); Test group 1: TMR + CPs (1.0 g·kg^−1^); Test group 2: TMR + CPs (2.0 g·kg^−1^); Test group 3: TMR + CPs (3.0 g·kg^−1^).

## Data Availability

The data presented in this study are available upon request from the corresponding authors.
